# Histological Diagnosis and Recovery Regulation of Gametophyte Developmental Disorders During In Vitro Propagation of the Medicinal Fern *Cibotium barometz*

**DOI:** 10.3390/plants15172570

**Published:** 2026-08-24

**Authors:** Wumei Si, Yunfang Zhang, Heng Jiang, Jingyi Yuan, Kunhua Wei, Quan Yang, Gang Xu

**Affiliations:** 1 School of Traditional Chinese Medicine, Guangdong Pharmaceutical University, Guangzhou 510006, China; swm15556503812@163.com (W.S.); yuanjingyi0071@163.com (J.Y.); divinekh@163.com (K.W.); 2 School of Pharmacy, Guangdong Pharmaceutical University, Guangzhou 510006, China; 16696379526@163.com (Y.Z.); jh15874556538@163.com (H.J.); 3 Key Laboratory of Lingnan Medicinal Materials Production and Development, National Administration of Traditional Chinese Medicine, Guangzhou 510006, China; 4 Guangzhou Comprehensive Experimental Station, China Agriculture Guangzhou Research System for Chinese Medicinal Materials, Guangzhou 510006, China; 5 Guangdong Engineering Technology Center for Planting and Comprehensive Development of Lingnan Traditional Chinese Medicinal Materials, Guangzhou 510006, China

**Keywords:** *Cibotium barometz*, archegonial ontogeny, gametophyte disorder, histology, in vitro propagation

## Abstract

*Cibotium barometz* (L.) J.Sm., a medicinal fern, relies on three interconnected processes for in vitro propagation: spore germination, ordered gametophyte development, and successful initiation and early establishment of the sporophyte generation. However, during aseptic subculture, gametophytes frequently deviate from this developmental trajectory. The predominant abnormalities include filamentous arrest, thick fan-shaped blades, and irregular spatulate blades, all of which were associated with reduced archegonial maturation and subsequent sporophyte establishment. In this study, developmental time-course observation, paraffin-section histology, and medium-regulation experiments were integrated to establish a diagnosis–recovery–initiation framework for *Cibotium barometz* gametophytes. Spore germination and gametophyte development were divided into eight practical checkpoints: spore imbibition, spore-wall rupture, rhizoid emergence, rhizoid elongation, filamentous prothallus, lamellar prothallus, cordate gametophyte, and sporophyte formation. The ontogeny of archegonia was delineated into six histological stages, ranging from the initial-cell stage to the pre-fertilization stage. Based on an integrative assessment of population appearance, individual morphology, and anatomical sections, abnormal gametophytes were classified into three diagnostic categories. Filamentous arrest manifested as wool-like or thread-like growth patterns, featuring persistent chain-like cellular organization and failure to develop a two-dimensional blade. Thick fan-shaped blades showed pronounced enlargement and thickening; section images revealed narrow, densely packed cells, accumulation of storage compounds, and localized wall thickening, a pattern consistent with, but not sufficient to demonstrate, a preferential shift toward vegetative proliferation. Irregular spatulate blades were narrow and asymmetric and showed morphological and histological features suggestive of disrupted tissue polarity. Although archegonial initial cells or primordium-like structures were intermittently detected, they infrequently advanced to mature archegonia. Medium treatment significantly affected arrest rate, hypertrophy rate, sporophyte initiation, and mean young-sporophyte height (*p* < 0.001). Murashige and Skoog (MS) medium supplemented with IAA, 6-BA, and activated carbon was associated with the highest arrest rate, whereas full-strength MS medium was associated with the most pronounced hypertrophy. Among hormone-free subculture treatments, 1/2 MS + activated carbon gave the highest sporophyte initiation rate (71.45% ± 9.16%); the same formula also performed well in primary hormone-free cultures (70.54% ± 9.07%). Correlation analysis showed a strong positive association between sporophyte initiation rate and mean young-sporophyte height (*r* = 0.935, *p* < 0.001), while arrest and hypertrophy were negatively associated with sporophyte initiation. These results support evaluating in vitro propagation using quantified developmental outcomes—arrest rate, hypertrophy rate, sporophyte initiation rate, and mean young-sporophyte height—rather than biomass alone. Under the tested conditions, hormone-free 1/2 MS + activated carbon was associated with the most favorable recovery outcomes; the underlying mechanisms require validation by quantitative histology, gene-expression analysis, endogenous-hormone profiling, and direct measurement of compounds adsorbed by activated carbon.

## 1. Introduction

*Cibotium barometz* (L.) J.Sm., a member of Cibotiaceae, is valued as a medicinal plant, an ornamental fern and a germplasm resource. Taxonomic databases, chloroplast-genome studies, phylogenomic analyses, and chromosome-level genomic work have provided an increasingly firm basis for its classification and utilization [1,2,3,4,5]. The rhizome serves as the traditional medicinal component; however, rhizome extraction induces plant mortality or substantial injury, thereby compromising natural population regeneration. Consequently, the establishment of a dependable in vitro and nursery propagation system is essential to mitigate pressure on wild resources [6,7].

The reproductive process in ferns is distinguished from that of seed plants owing to the independent life cycle of the gametophyte generation. The gametophyte is not merely a transient green tissue; rather, it plays a decisive role in the formation of archegonia and antheridia, the potential for fertilization, and the establishment of the sporophyte [8,9,10,11,12,13,14,15]. In homosporous ferns, gametophyte development typically encompasses the establishment of polarity, the transition from a filamentous to a lamellar structure, the formation of a meristematic region, the development of a cordate notch, the differentiation of gametangia, and the initiation of the young sporophyte [8,9,10,11,12,13,14]. Therefore, a culture system that generates abundant green tissue cannot be considered efficient if the tissue exhibits unstable polarity or fails to develop functional reproductive organs.

Previous studies have demonstrated that spores of *Cibotium barometz* are capable of germinating under aseptic conditions, with cordate gametophytes typically emerging within approximately 45–61 days. The composition of the culture medium significantly influences both spore germination and gametophyte development [16,17,18]. Subsequent research has further optimized protocols for prothallus proliferation, sporophyte initiation, and rapid propagation via tissue culture [19,20]. However, under experimental conditions, three distinct abnormal gametophyte phenotypes are commonly observed. Certain specimens persist in a filamentous or tomentose state for extended periods, failing to develop two-dimensional prothalli; others form large, thickened, fan-shaped, or aggregated blade structures; and a subset exhibits irregularly spatulate or linguiform morphologies. Categorizing all such anomalies under the broad labels of “poor growth” or “deformity” obscures the underlying developmental constraints and impedes targeted optimization of culture media.

The ionic strength of the medium, the inclusion of activated carbon, and the application of exogenous plant growth regulators are important components of the in vitro microenvironment. Murashige and Skoog (MS) salts and their reduced-strength derivatives, activated carbon, and plant growth regulators may influence ionic strength, osmotic conditions, phenolic-compound accumulation, and hormone-related responses [7,21,22,23,24,25,26,27,28,29]. Activated carbon is commonly used to adsorb phenolics, metabolic inhibitors, and residual plant growth regulators, although the compounds actually adsorbed and the resulting physiological effects are context-dependent [22,23,24]. Auxins, cytokinins, and gibberellins regulate cell division, organogenesis, sexual expression, and apogamy [29,30,31,32,33,34,35,36,37,38]. A hormone combination that benefits vegetative proliferation could therefore be associated with altered polarity maintenance or archegonial maturation after the gametophyte enters reproductive differentiation; however, causal inference requires endogenous-hormone and molecular validation.

On this basis, this study combined data from spore-germination timing, archegonial ontogeny, histological diagnosis of developmental disorders, and medium-based recovery treatments. The aim was to construct a practical diagnosis–recovery–initiation framework for gametophyte disorders in *Cibotium barometz*, characterize the morphological and histological differences among abnormal phenotypes, and identify culture conditions associated with improved developmental outcomes. Rather than treating biomass increase as the sole criterion, the study used quantified rates of arrest, hypertrophy, and sporophyte initiation, together with mean young-sporophyte height. Because endogenous hormones, gene expression, and compounds adsorbed by activated carbon were not measured, the proposed physiological mechanisms are considered working hypotheses rather than demonstrated causal pathways.

## 2. Results

### 2.1. Developmental Checkpoints from Spore Germination to Sporophyte Initiation

**Figure 1:**
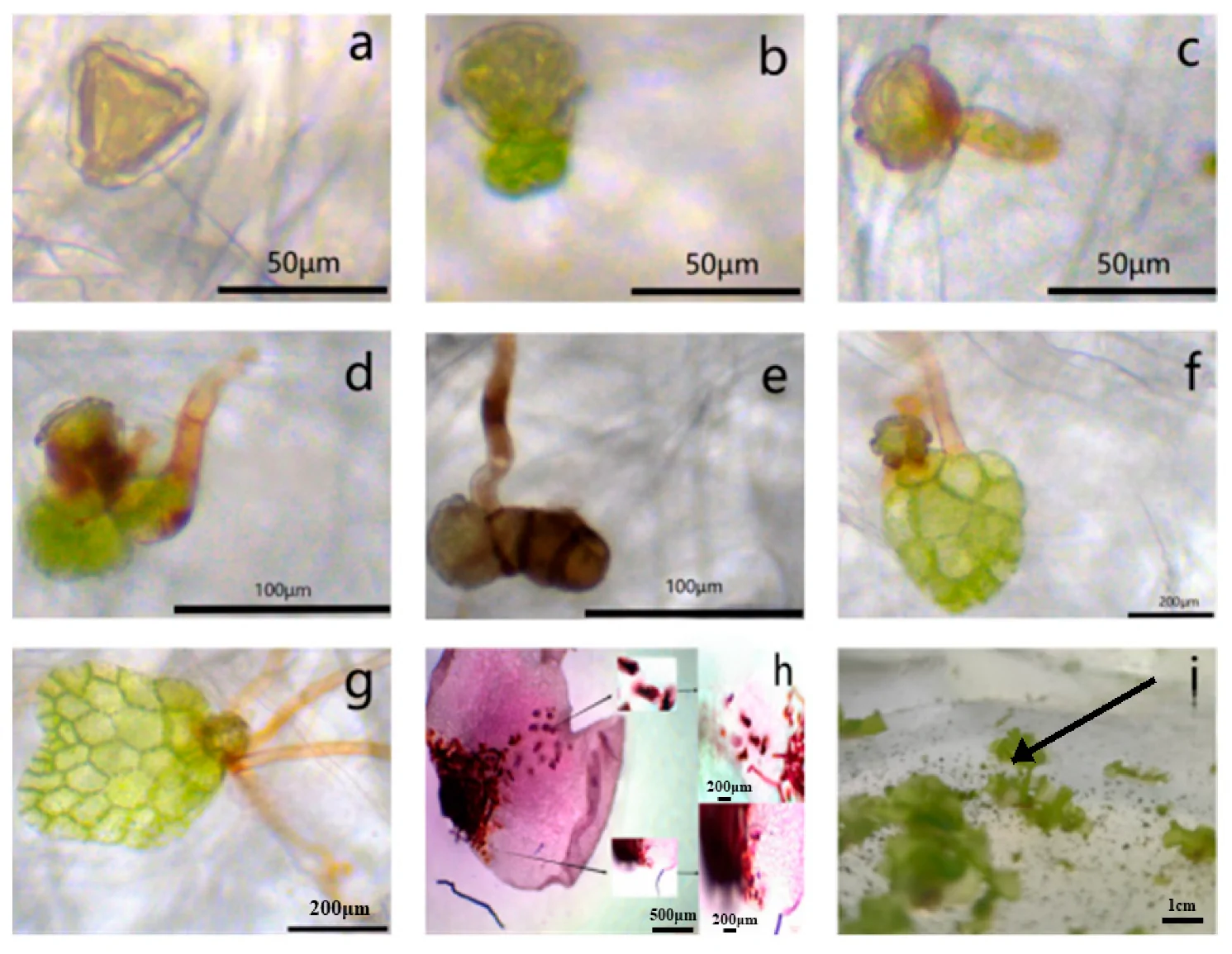
Developmental stages from spore germination to sporophyte formation in *Cibotium barometz*. (**a**) spore imbibition; (**b**) spore-wall rupture; (**c**) rhizoid emergence; (**d**) rhizoid elongation; (**e**) filamentous prothallus; (**f**) lamellar prothallus; (**g**) cordate gametophyte; (**h**) gametangial region; (**i**) young sporophyte. In panel (**i**), the arrow indicates the emerging young sporophyte.

**Figure 2:**
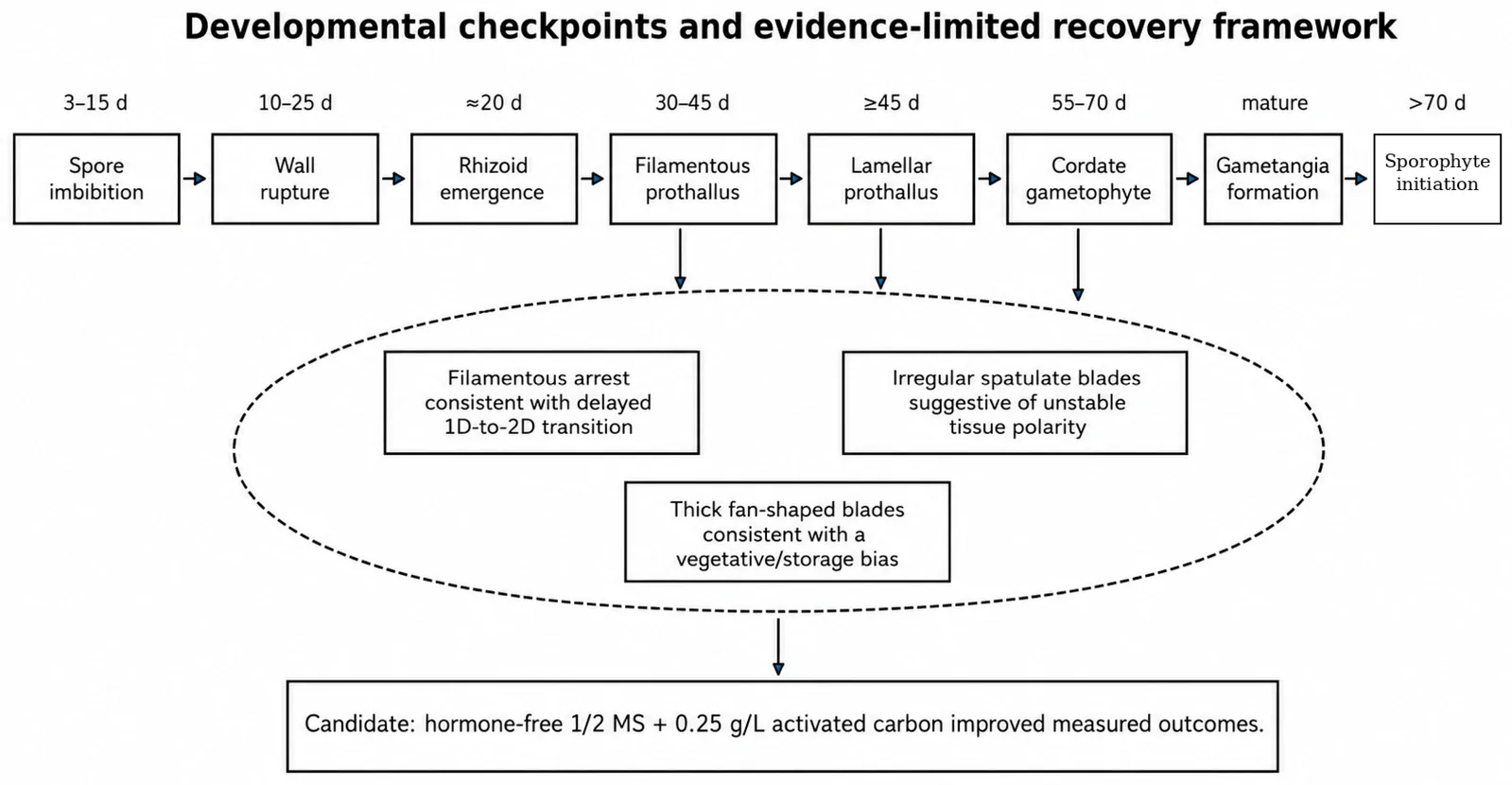
Developmental checkpoints and evidence-limited recovery framework for *Cibotium barometz* gametophytes. MS, Murashige, and Skoog medium. Note: The scheme summarizes the normal developmental sequence, the occurrence windows of the three disorder types, and the candidate recovery condition of hormone-free one-half-strength MS (1/2 MS) medium supplemented with 0.25 g/L activated carbon.

Under aseptic culture conditions, *Cibotium barometz* exhibited a relatively consistent developmental sequence following inoculation (Figure 1 and Figure 2; Table 1). Within the initial 3–15 days, spores imbibed water and underwent swelling. The outer wall of the trilete spores remained intact, with no observable rupture. Microscopic examination revealed that hydrated spores exhibited a more rounded morphology, indicative of a preparatory stage characterized by water uptake and reserve mobilization prior to true germination (Figure 1a). Germination commenced at 10–25 days, marked by the appearance of distinct fissures or apertures in the spore wall. As the rupture expanded, the germinating cell began to protrude through the spore-wall aperture while remaining bounded by its own cell wall; no filamentous extension had yet developed. This phase marked the initiation of germination (Figure 1b). Approximately 20 days after inoculation, a unicellular filamentous structure, designated as the primary rhizoid, emerged from the wall opening. A limited number of chloroplasts were observed within the rhizoid, suggesting early physiological activity (Figure 1c). Between 20 and 40 days, the rhizoid transitioned from a short unicellular tube to an elongated structure. The rhizoid system progressively expanded from a solitary filament into a network, thereby providing physical anchorage and an initial absorptive surface for the juvenile gametophyte (Figure 1d). At 30–45 days, the growth pattern underwent a transition. Linear filamentous growth began to give way to lamellar expansion; one to three smooth, single-row somatic cells emerged on the same side as the rhizoids, representing the earliest lamellar initials. The rhizoid network maintained its initial configuration, while the upper portion of the organism transitioned from a filamentous to a blade-like morphology (Figure 1e). Beyond 45 days, cell division distinctly shifted from a single-row pattern to lamellar expansion. Leaf-like discs became discernible, and repeated two-dimensional divisions generated a flat, rapidly expanding prothallus (Figure 1f). At 55–70 days, cells at the apex and both lateral margins underwent active division, the margin curved inward, and a symmetrical cordate gametophyte developed. At this stage, the notch, wings, midrib-like central region, and clustered ventral rhizoids became apparent (Figure 1g). Upon establishment of the prothallus architecture, the tissue initiated sexual differentiation and produced archegonia and antheridia (Figure 1h). In the presence of a free water film, spermatozoids swam into archegonia and fertilization occurred. The zygote developed within the venter into a multicellular embryo, which then differentiated into a primary root, an upward-growing axis, and the first young leaf, often with a circinate form (Figure 1i). Continued cell proliferation and expansion allowed the young sporophyte to break through the gametophyte and establish an independent autotrophic system. This stage completed the transition from the gametophyte generation to the sporophyte generation.

The early checkpoints—spore imbibition, wall rupture, and rhizoid emergence—are suitable for scoring germination. The middle phase is defined by the transition from a filamentous prothallus to a lamellar prothallus, while the later phase is marked by the cordate gametophyte, gametangium formation, and sporophyte emergence. The filament-to-lamina transition represents the first major developmental-quality checkpoint. When cells remain in one-dimensional chain-like division for too long, filamentous arrest or piled cell clusters are likely to form. This developmental sequence corroborates prior investigations into the in vitro development of *Cibotium barometz* and conforms to the fundamental principles of fern gametophyte morphogenesis [8,9,10,11,12,13,14,16,17,18].

### 2.2. Antheridial Differentiation and Archegonial Ontogeny

**Figure 3:**
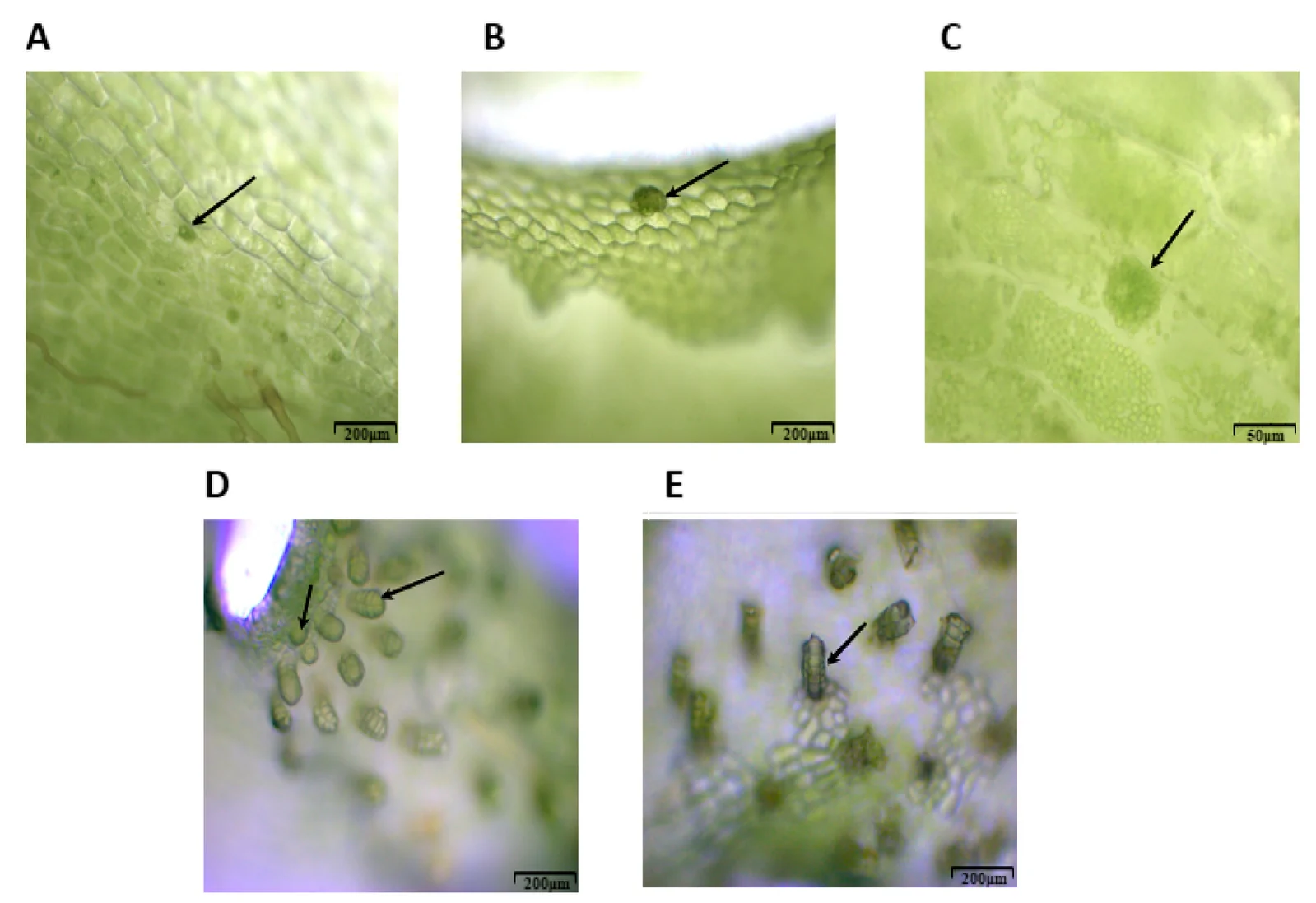
Microscopic observation of antheridia and archegonia in *Cibotium barometz*. (**A**) early antheridium; (**B**) mature antheridium; (**C**) antheridium releasing sperm cells; (**D**) early archegonium and early neck-cell differentiation; (**E**) late neck-cell differentiation. Note: Arrows indicate the representative antheridial or archegonial structures described in the corresponding panels.

Microscopic observation showed that antheridia developed from early rounded structures into mature antheridia, which subsequently released sperm cells (Figure 3A–C).

**Figure 4:**
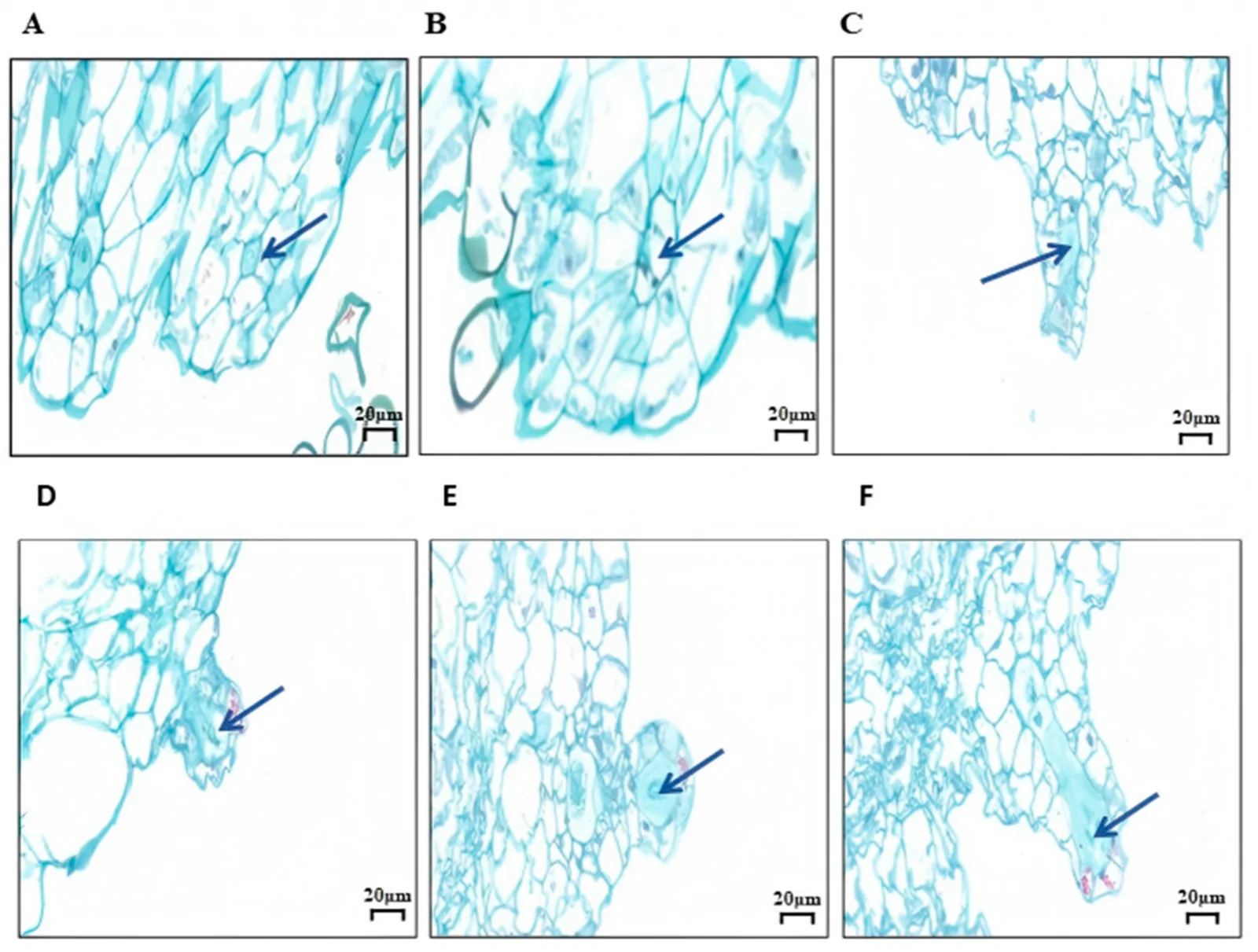
Paraffin-section observation of archegonial ontogeny in *Cibotium barometz*. (**A**) initial-cell stage; (**B**) primordium stage; (**C**) early neck-cell differentiation; (**D**) late neck-cell differentiation; (**E**) mature stage; (**F**) pre-fertilization stage. Note: Arrows indicate the archegonial structures corresponding to the developmental stage shown in each panel.

Archegonia originated from local initial cells and early primordia, followed by differentiation of neck cells and formation of a visible neck structure (Figure 3D,E). Paraffin sections allowed archegonial ontogeny to be divided into six stages: initial-cell stage, primordium stage, early neck-cell differentiation, late neck-cell differentiation, mature stage, and pre-fertilization stage (Figure 4; Table 2). At the initial-cell stage, obvious reproductive structures were absent; cells were mostly polygonal or irregular and were arranged rather loosely (Figure 4A). At the primordium stage, some cells aggregated locally and underwent directional division, producing a compact archegonial primordium (Figure 4B). During early neck-cell differentiation, polarity became evident within the primordium. Neck cells began to elongate and align longitudinally, the venter region expanded, and the archegonium acquired an early flask-like outline (Figure 4C). In the late stage of neck-cell differentiation, the neck cells exhibited a more regular arrangement, the tubular neck became more distinct, and the egg cell and ventral canal cell within the venter were more readily discernible (Figure 4D). Upon reaching maturity, a characteristic flask-shaped archegonium was observed, featuring a slender neck, an enlarged venter, and a distinguishable egg cell, suggesting potential fertilization capability (Figure 4E). During the pre-fertilization stage, the neck canal was clearly defined, and the egg cell exhibited intense staining; degeneration of canal cells likely commenced, thereby establishing an entry pathway for sperm cells (Figure 4F). Together, these observations describe the gradual transformation of vegetative gametophyte cells into functional female reproductive organs.

### 2.3. Histological Diagnosis of Developmental Disorder Types

**Figure 5:**
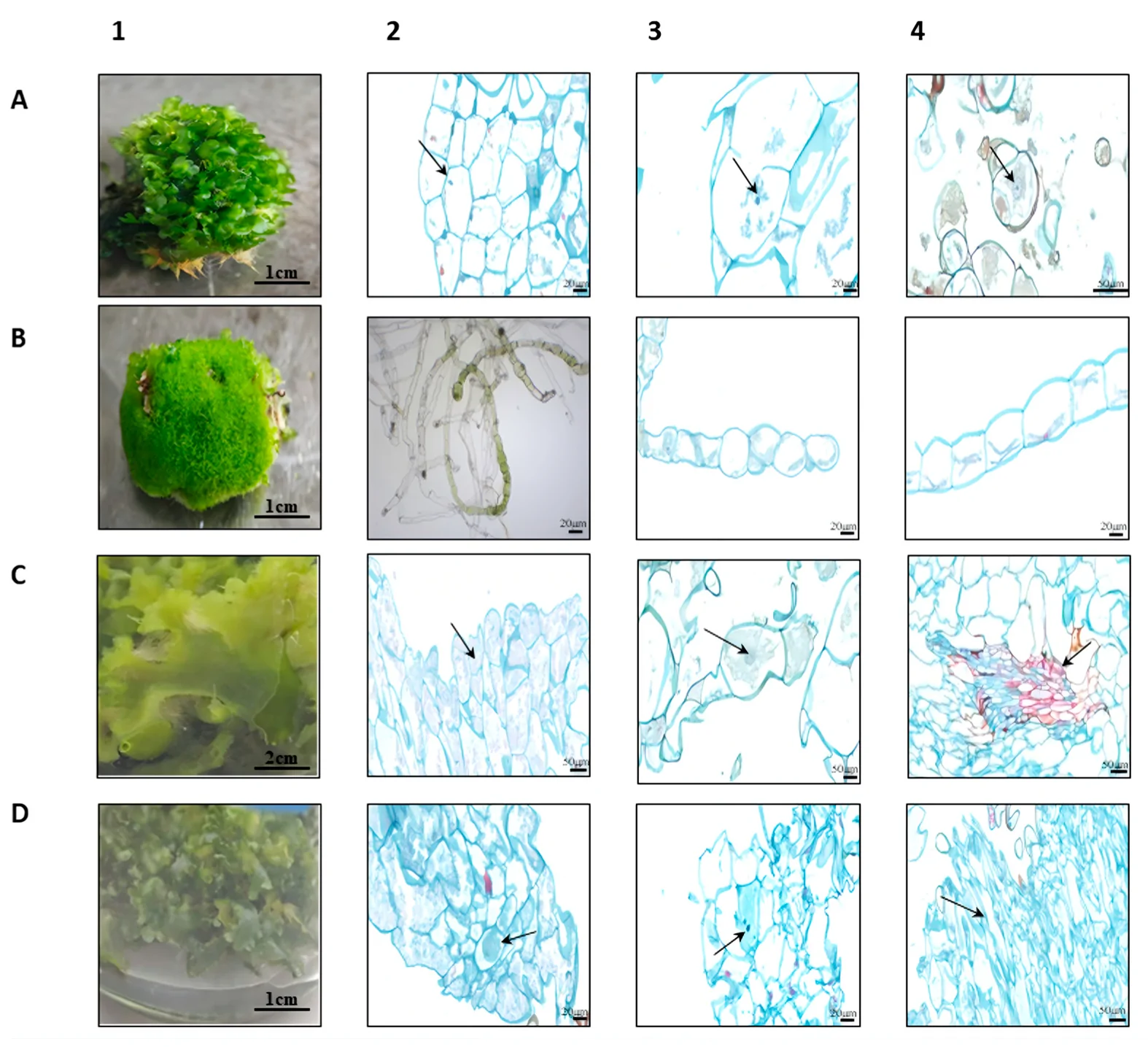
Paraffin-section microstructure of normal and developmentally disordered gametophytes. Note: Rows (**A**–**D**) represent normal prothalli, filamentous arrest, thick fan-shaped blades, and irregular spatulate blades, respectively. Column (**1**) shows external morphology; columns (**2**–**4**) show representative paraffin-section images. Arrows indicate representative diagnostic structures or abnormalities described in the text, including ordered cell boundaries/nuclei in normal prothalli, persistent filamentous organization in filamentous arrest, densely packed or locally thickened tissues in thick fan-shaped blades, and archegonial initials or abnormal cytological features in irregular spatulate blades.

Based on population morphology and paraffin-section anatomy, abnormal *Cibotium barometz* gametophytes were grouped into three types: filamentous arrest, thick fan-shaped blades, and irregular spatulate blades (Table 3). Normal prothalli, used as the control, were green and flat, with a regular lamellar or nearly cordate outline. Their margins were clear, and the central region gradually thickened to form wing-like tissue (Figure 5(A-1)). Sections showed ordered cell arrangement, distinguishable epidermal and inner parenchymatous cells, intact cell boundaries (Figure 5(A-2)), and relatively clear nuclei (Figure 5(A-3,A-4)). At maturity, archegonial primordia or more developed archegonia were observed, consistent with completion of the transition from filamentous growth to a two-dimensional blade and with histological competence for reproductive differentiation. In contrast, gametophytes with filamentous arrest were usually wool-like or thread-like in overall appearance (Figure 5(B-1)). These gametophytes displayed a coloration ranging from light green to green, with indistinct individual outlines and lacking the marginal expansion typical of laminar prothalli. Microscopic analysis indicated that their structure was primarily composed of unidirectional cell filaments, characterized by continuous division and elongation along a single axis. Although loose, interwoven bundles occasionally formed in localized areas, a two-dimensional blade resulting from a plane-shift in cell division was not observed (Figure 5(B-2)). Sectional analysis further confirmed a simplified organizational architecture, dominated by a unidirectional cell arrangement, minimal cellular stratification, absence of a distinct central wing-like region, and no stable archegonial primordia or mature gametangia (Figure 5(B-3,B-4)). In contrast, thick fan-shaped gametophytes exhibited significant enlargement and thickening, frequently developing into broad fan-shaped, clumped, or irregular blades with a darker green surface and increased local tissue density (Figure 5(C-1)). Although these samples had greater biomass and larger blade area than normal cordate prothalli, their margins were irregular and the central notch or growth-point region was poorly defined. Single-body observation revealed thick blades, local bulging, and reduced transparency, all suggesting excessive internal cell accumulation. Paraffin sections showed that many cells were narrow or compressed, closely packed, and separated by small intercellular spaces. Local storage accumulation was common; some walls were thickened and even showed staining patterns suggestive of local lignification or senescence (Figure 5(C-2,C-4)). In some cells, the nuclear membrane was blurred (Figure 5(C-3)). These observations indicate that the phenotype was not characterized by a simple lack of growth and are consistent with excessive vegetative expansion or altered tissue differentiation; however, the underlying mechanism was not tested. The larger blade and greater thickness were not accompanied by greater reproductive output and may have been associated with reduced archegonial primordium formation, antheridial differentiation, or fertilization-space establishment; the proposed roles of overproliferation, storage accumulation, and tissue compaction remain to be tested quantitatively. Irregular spatulate gametophytes were generally narrow, asymmetric, and spoon- or tongue-shaped. Some samples were locally swollen at the base or apex; both sides developed unevenly, and the normal cordate outline was indistinct (Figure 5(D-1)). Individual morphology showed that these samples had partially acquired lamellar expansion, but the direction of expansion was unstable. Edge cells divided unevenly, and protrusions, bends, or distorted extensions appeared in local regions. Compared with filamentous-arrest materials, irregular spatulate blades had partly completed the one-dimensional to two-dimensional transition; compared with normal cordate prothalli, however, their apical growth region, central wing-like tissue, and bilateral symmetry had not stabilized. Sections revealed disordered internal cell arrangement and unclear tissue polarity (Figure 5(D-4)). Archegonial initial cells or primordium-like structures were observed in some areas (Figure 5(D-2)), indicating that reproductive differentiation could still be initiated. Nevertheless, these structures were unevenly distributed, and later neck and venter differentiation was insufficient for the formation of mature archegonia. In some cells, blurred nuclear membranes, abnormal nuclear organization, or multinucleate-like features were also present (Figure 5(D-3)), which are features compatible with, but not direct evidence of, altered cell division, cell-fate maintenance, or tissue-polarity regulation.

### 2.4. Effects of Medium Treatment on Developmental Recovery and Sporophyte Initiation

**Figure 6:**
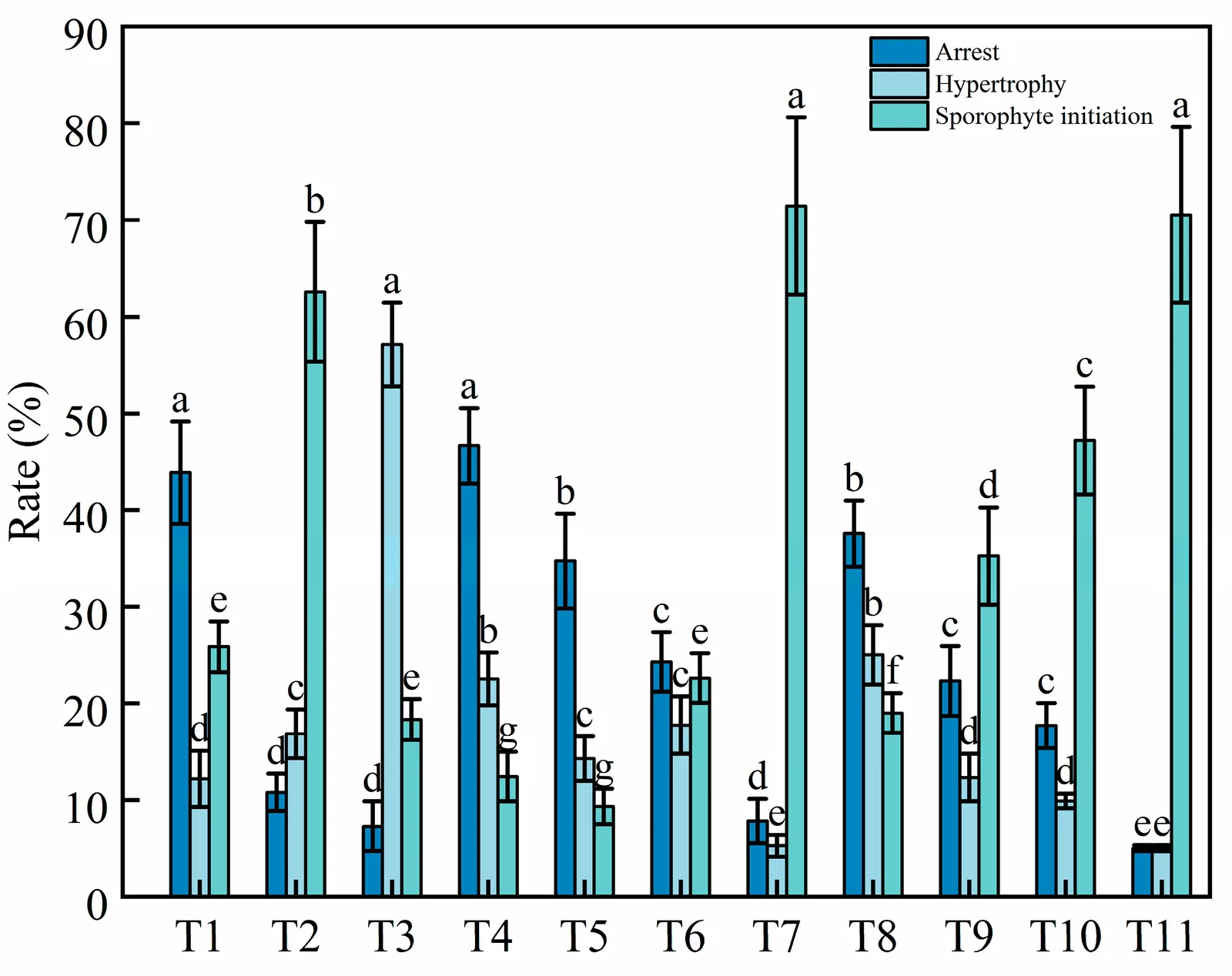
Effects of representative media treatments on arrest rate, hypertrophy rate, and sporophyte initiation rate. Note: Bars show means and error bars show standard deviations. Different lowercase letters indicate significant differences among treatments at *p* < 0.05.

Medium treatment had highly significant effects on all four developmental indicators (Table 4; Figure 6). One-way ANOVA showed significant treatment effects on arrest rate (F = 225.36, *p* = 9.08 × 10^−20^), hypertrophy rate (F = 247.76, *p* = 3.24 × 10^−20^), sporophyte initiation rate (F = 666.14, *p* = 6.63 × 10^−25^), and mean young-sporophyte height (F = 1031.65, *p* = 5.49 × 10^−27^). Kruskal–Wallis tests gave consistent results (all *p* ≤ 0.000522), supporting the robustness of the treatment differences. In the subculture + hormone group, full-strength MS medium supplemented with IAA, 6-BA, and activated carbon was associated with the highest arrest rate (43.88% ± 5.30%). Because this treatment combined full-strength salts, two plant growth regulators, and activated carbon, and because endogenous hormone levels were not measured, the data do not demonstrate that auxin-cytokinin input caused developmental arrest. In contrast, 1/2 MS medium supplemented with GA_3_ and 6-BA was associated with a sporophyte initiation rate of 62.56% ± 7.23%; the respective contributions of reduced salt strength and the two regulators cannot be separated in this design. Full-strength MS medium alone was associated with the highest hypertrophy rate (57.12% ± 4.34%), a pattern consistent with, but not proof of, a nutrient-related vegetative bias. Among hormone-free subculture materials, 1/2 MS medium supplemented with 0.25 g/L activated carbon achieved the highest sporophyte initiation rate (71.45% ± 9.16%) while maintaining low arrest and hypertrophy. The same formulation also showed favorable performance in primary hormone-free materials, with a sporophyte initiation rate of 70.54% ± 9.07% and a mean young-sporophyte height of 4.16 ± 0.72 cm.

### 2.5. Intercorrelations of Developmental Indicators

**Figure 7:**
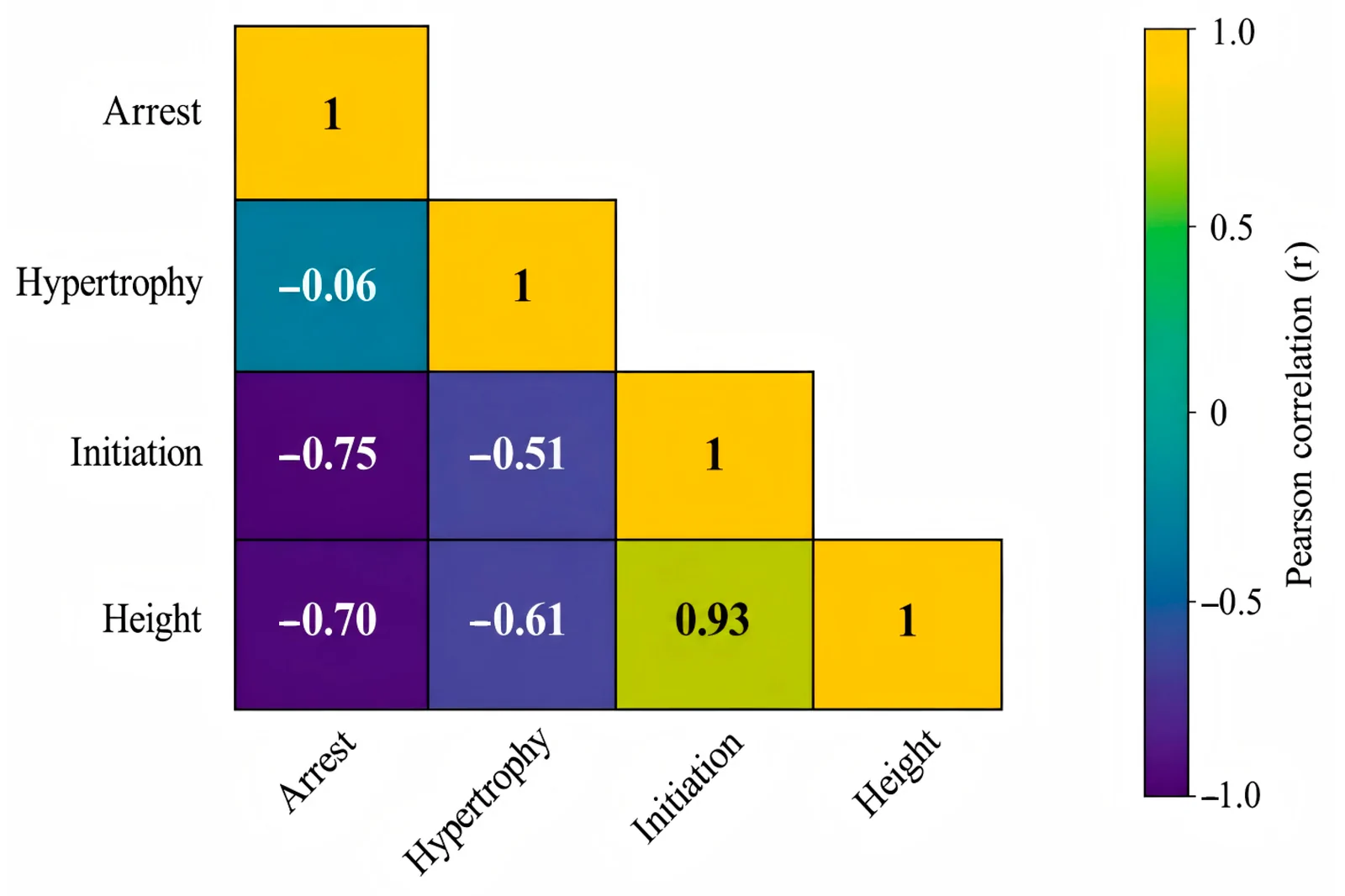
Lower-triangular Pearson correlation heat map of developmental indicators. Labels use “Initiation” for sporophyte initiation rate and “Height” for mean young-sporophyte height. Note: Arrest rate and hypertrophy rate were negatively correlated with sporophyte initiation and young-sporophyte height, whereas sporophyte initiation rate was strongly positively correlated with young-sporophyte height. Correlations describe association and do not demonstrate causality.

Correlation analysis described the co-variation among developmental indicators (Figure 7). Sporophyte initiation rate exhibited a highly significant positive correlation with mean young-sporophyte height (Pearson *r* = 0.935, *p* = 1.68 × 10^−15^). Arrest rate was negatively correlated with sporophyte initiation rate (*r* = −0.752, *p* = 4.57 × 10^−7^), and hypertrophy rate was also negatively correlated with sporophyte initiation rate (*r* = −0.505, *p* = 0.00272). These associations show that treatments with lower arrest and hypertrophy tended to have higher sporophyte initiation and juvenile growth, but correlation does not establish the developmental, hormonal, or metabolic mechanisms responsible.

## 3. Discussion

### 3.1. Developmental Quality Can Be Operationalized by Measured Outcomes Rather than Biomass Alone

A major implication of this study is that *Cibotium barometz* gametophyte culture can be evaluated by developmental performance rather than biomass accumulation alone. In this experiment, developmental quality was operationalized using four quantified outcomes: arrest rate and hypertrophy rate as adverse indicators, and sporophyte initiation rate and mean young-sporophyte height as favorable indicators. No composite developmental-quality score was calculated. Large green gametophyte clumps may appear vigorous but do not necessarily show reproductive competence. Full-strength Murashige and Skoog (MS) medium was associated with a high proportion of thick fan-shaped blades and a low sporophyte initiation rate. Fern gametophyte development normally involves an ordered transition from a one-dimensional cell filament to a two-dimensional blade, followed by meristem establishment, cordate-notch formation, and differentiation of functional gametangia [8,9,10,11,12,13,14]. These data are therefore consistent with the possibility that greater nutrient availability can favor vegetative expansion without a corresponding improvement in reproductive output, although the cellular and molecular basis of this pattern was not tested.

### 3.2. The Three Disorder Types Are Associated with Different Putative Developmental Bottlenecks

Filamentous arrest is morphologically consistent with failure or delay of the one-dimensional-to-two-dimensional transition. This transition depends on changes in the plane of cell division and establishment of a multicellular meristematic region, which together convert a chain-like filament into a cordate prothallus [9,10,11,12,13,14]. Materials that remain chain-like or wool-like for an extended period also showed poor formation of the cordate notch, wing-like tissue, and archegonia. Thick fan-shaped blades represent a distinct morphological deviation. Their increased tissue volume, storage-like accumulation, and localized wall thickening are compatible with a vegetative/storage bias or localized senescence rather than efficient reproductive differentiation; these interpretations require morphometric, histochemical, and molecular confirmation. A useful in vivo contrast is provided by reports of *Cibotium barometz* gametophytes developing in natural media [17]. Natural substrates generally supply minerals more gradually and contain organic and mineral surfaces that can buffer or adsorb dissolved compounds, whereas full-strength artificial medium provides a comparatively concentrated and immediately available nutrient environment. Thus, the persistence of filamentous or hypertrophic phenotypes under some in vitro conditions may reflect, at least in part, a mismatch between the artificial microenvironment and the ecological conditions under which fern gametophytes normally develop. This comparison is intentionally speculative because nutrient availability and sorption properties of the natural substrate were not measured in this study.

Irregular spatulate blades warrant particular attention because some retained archegonial initial cells or primordium-like structures. This observation suggests that reproductive differentiation can begin but may not be stably maintained. Compared with completely arrested filamentous materials, irregular spatulate blades may therefore retain greater recovery potential, but this inference should be tested by lineage tracking and quantitative sporophyte initiation experiments. Distinguishing filamentous arrest, thick fan-shaped blades, and irregular spatulate blades nevertheless provides reproducible working categories for material selection, timing of recovery culture, and design of subsequent validation experiments.

### 3.3. Improved Performance on Low-Salt Activated-Carbon Medium Does Not Establish an Adsorption Mechanism

Hormone-free 1/2 MS supplemented with 0.25 g/L activated carbon was associated with the most favorable combination of low arrest, low hypertrophy, high sporophyte initiation, and greater mean young-sporophyte height in both subculture and primary materials. Low-salt culture could reduce ionic or osmotic stress, and activated carbon could adsorb phenolic compounds, metabolic inhibitors, residual plant growth regulators, or other metabolites, as reported in other plant tissue-culture systems [16,19,20,22,23,24,25,26,27,28,29]. However, this study did not quantify medium osmolarity, tissue ion status, phenolic compounds, residual hormones, toxic metabolites, or their concentrations before and after contact with activated carbon. Consequently, the data demonstrate a treatment association but do not identify which, if any, adsorption or stress-alleviation mechanism was responsible.

The poorer performance of MS + activated carbon relative to 1/2 MS + activated carbon suggests that the outcome of activated-carbon supplementation depends on the basal medium. Nevertheless, salt strength and activated carbon were not examined in a fully factorial dose-response design, and activated carbon can also adsorb beneficial nutrients or regulators. These findings therefore support hormone-free 1/2 MS + 0.25 g/L activated carbon as a candidate recovery treatment under the tested conditions, rather than as a universal promoter or a demonstrated microenvironment-resetting agent. From an ecological perspective, the favorable response to 1/2 MS plus activated carbon can be viewed as partially reproducing two broad features of many natural fern substrates—lower nutrient intensity and greater chemical buffering or sorption capacity—rather than as evidence that activated carbon is a direct natural analog of soil organic matter. The hypothesis should be tested by directly comparing nutrient concentrations, dissolved phenolics, or other metabolites, and sorption behavior in the culture media and representative natural substrates [7,17,23].

### 3.4. Associations with Exogenous Plant Growth Regulators Require Endogenous-Hormone Validation

The high arrest rate observed in the combined MS + IAA + 6-BA + activated-carbon treatment is an association among several bundled treatment components. Auxin and cytokinin are central regulators of cell division, organ formation, and in vitro regeneration [29,34,35,36,37], and plant growth regulators can influence sex expression, apogamy, and sporophyte initiation in ferns [30,31]. However, endogenous auxin, cytokinin, and gibberellin concentrations, hormone uptake, and hormone stability in the medium were not measured. This experiment therefore cannot demonstrate that auxin/cytokinin treatment induced developmental arrest or that hormonal imbalance caused the observed phenotypes. Such statements should be regarded as hypotheses for future testing.

Similarly, the relatively high sporophyte initiation rate in the 1/2 MS + GA_3_ + 6-BA treatment does not establish that the two regulators promoted sporophyte initiation, because reduced salt strength and hormone supplementation were applied together. Gibberellin-related signaling and antheridiogen systems are relevant to fern sexual expression [30,33], but their involvement in *Cibotium barometz* requires direct evidence. Factorial experiments varying salt strength, regulator identity, concentration, and exposure duration, combined with endogenous-hormone profiling and expression analysis of hormone-response and developmental-regulator genes, are needed before stage-specific hormonal recommendations can be made.

### 3.5. Practical Significance and a Testable Diagnosis–Recovery–Initiation Workflow

The practical value of this study lies in linking visible disorder phenotypes to quantified culture outcomes. For routine propagation under the conditions tested, the simplest practical recommendation is to use hormone-free 1/2 MS medium supplemented with 0.25 g/L activated carbon as the first-choice recovery and sporophyte initiation medium, because this formulation produced the lowest adverse developmental indices and the highest sporophyte initiation rate among the tested hormone-free treatments. Materials can first be classified morphologically and qualitatively by histological examination as normal prothalli, filamentous arrest, thick fan-shaped blades, or irregular spatulate blades; abnormal materials can then be transferred directly to this formulation and monitored for restoration of regular lamellar or cordate morphology, mature or nearly mature archegonia, and subsequent sporophyte initiation. This recommendation is empirical rather than mechanistic and is limited to the tested culture system. Before large-scale application, its reproducibility should be evaluated across genotypes, culture batches, acclimatization conditions, and nursery environments.

### 3.6. Limitations and Future Research Directions

The principal limitation is that the study is descriptive and associational rather than mechanistic. The histological analysis was used to define qualitative diagnostic categories, but cell area, blade thickness, cell density, intercellular-space proportion, frequency of nuclear abnormalities, and density of storage granules were not measured. Future studies should apply blinded image analysis to serial sections from independent biological replicates and quantify these variables with predefined regions of interest. Such morphometric data would allow the diagnostic categories and the concept of developmental quality to be evaluated more objectively.

Molecular and hormonal validation is also lacking. No transcriptomic analysis or targeted gene-expression assay was performed for regulators related to cell-division orientation, meristem identity, polarity establishment, gametangial differentiation, or auxin/cytokinin/gibberellin signaling. Endogenous IAA, cytokinins, and gibberellins were not quantified. Therefore, the proposed links among exogenous regulators, polarity disturbance, reproductive differentiation, and developmental arrest remain hypotheses. RNA sequencing followed by qRT-PCR validation, together with stage-matched endogenous-hormone profiling, would be required to determine whether the phenotypes share common regulatory changes or represent distinct developmental states.

The mechanism of activated carbon was not examined directly. Phenolic compounds, residual plant growth regulators, and potentially toxic metabolites were not measured in media before and after activated-carbon treatment, and adsorption capacity was not tested. A factorial experiment separating salt strength from activated-carbon concentration, accompanied by chemical analysis of the medium and tissue, is needed to distinguish adsorption, nutrient modification, and osmotic effects. Finally, acclimatization survival, long-term sporophyte performance, and genetic stability should be incorporated into future validation. These experiments will clarify whether the candidate medium reverses an abnormal developmental state, selectively favors pre-existing competent cells, or acts through another process.

## 4. Materials and Methods

### 4.1. Plant Material, Spore Collection, Surface Disinfection, and Aseptic Culture

Mature spores were harvested in February 2023 from Rongshui, Guangxi Zhuang Autonomous Region, China. The collected material was taxonomically identified by Professor Gang Xu of Guangdong Pharmaceutical University as spores of *Cibotium barometz* (L.) J.Sm. A voucher specimen has been deposited at the School of Traditional Chinese Medicine, Guangdong Pharmaceutical University (specimen number: GXjmg2023002). Mature spore-bearing fronds were enclosed in clean paper bags and maintained under dry, ventilated conditions to facilitate natural spore release. After impurities were removed, spores were stored at 4 °C. For each disinfection batch, 10 mg of spore powder was suspended in 2 mL sterile water in a 15 mL centrifuge tube. A 5810 R centrifuge (Eppendorf SE, Hamburg, Germany) was used for centrifugation. After centrifugation at 3000× *g* for 3 min, the water was removed with a sterile pipette. The pellet was resuspended in 2 mL of 70% ethanol for 30 s and centrifuged again at 3000× *g* for 3 min, and the ethanol was removed. The spores were then treated with 2 mL of 5% NaClO for 8 min, centrifuged, rinsed five times with 2 mL sterile water, and finally resuspended in 2 mL sterile water. Ethanol and sodium hypochlorite used for surface disinfection were obtained from Sinopharm Chemical Reagent Co., Ltd. (Shanghai, China). Aliquots of 0.5 mL (2.5 mg spores) were spread evenly over 40 mL of solid one-eighth-strength Murashige and Skoog (1/8 MS) medium prepared from MS basal salts (M8522) and containing 7 g/L agar (A8190; Beijing Solarbio Science & Technology Co., Ltd., Beijing, China) in sterile 250 mL culture bottles; the spores were not immersed in liquid medium. Bottles were capped and incubated at 26 °C under a 12 h light/12 h dark photoperiod. 1/8 MS medium was used for the developmental time-course, whereas the recovery experiments used the solid media specified in Section 4.5. Subsequent experimental materials included aseptically cultured germinating spores, normal cordate prothalli, antheridia and archegonia at different developmental stages, developmentally disordered subculture gametophytes, and young sporophytes produced after medium treatments.

### 4.2. Developmental Time-Course Observation and Developmental Scoring

Beginning on the third day after inoculation, cultures were examined regularly for spore imbibition, spore-wall rupture, rhizoid emergence, rhizoid elongation, filamentous prothallus, lamellar prothallus, cordate gametophyte, gametangium formation, and sporophyte formation.Representative images were recorded using an SZX16 stereomicroscope and an IX73 inverted light microscope equipped with a DP75 digital camera (Evident Corporation, Tokyo, Japan). Developmental stages were assigned mainly according to morphology, with culture age used as supporting information. For each key stage, at least three independent culture bottles were examined, and no fewer than three representative fields were randomly selected from each bottle.

### 4.3. Paraffin Sectioning and Histological Observation

Normal cordate prothalli, developmentally disordered gametophytes, and archegonia at different stages were selected for paraffin-section observation. Samples were fixed immediately after collection in FAA fixative, prepared from 70% ethanol, formaldehyde, and glacial acetic acid at a volume ratio of 90:5:5. These analytical-grade reagents were obtained from Sinopharm Chemical Reagent Co., Ltd. (Shanghai, China). Fixation was carried out at room temperature for 24 h. For abnormal gametophytes with thickened blades or compact tissues, fixation was extended to 36 h to improve penetration. After fixation, samples were rinsed and stored in 70% ethanol before dehydration.

Fixed samples were dehydrated through an ethanol series: 70% ethanol for 30 min, 85% ethanol for 30 min, 95% ethanol for 30 min, absolute ethanol I for 30 min, and absolute ethanol II for 30 min. Xylene (Sinopharm Chemical Reagent Co., Ltd., Shanghai, China) was used as the clearing agent. Samples were first transferred to an absolute ethanol:xylene mixture (1:1, *v*/*v*) for 30 min and then to xylene I and xylene II for 30 min each until the tissues became translucent.

Cleared samples were infiltrated with paraffin and embedded. Paraffin with a melting point of 56–58 °C was used (Paraplast Plus, P3683; Sigma-Aldrich, St. Louis, MO, USA), and the infiltration temperature was maintained at 58–60 °C. Samples were successively transferred to paraffin I, II, and III for 45–60 min each. During embedding, the orientation of gametophyte blades and archegonial structures was adjusted to obtain suitable longitudinal or transverse sections. After solidification, the blocks were trimmed and serially sectioned with an RM2235 rotary microtome (Leica Microsystems Nussloch GmbH, Nussloch, Germany). The section thickness was 6–8 µm, with 7 µm used for routine observation. Sections were spread on warm water at 40–45 °C, mounted on glass slides, and dried overnight at 37 °C.

Sections were stained with the safranin-fast green double-staining method using plant staining reagents from the G1375 kit (Beijing Solarbio Science & Technology Co., Ltd., Beijing, China). After dewaxing in xylene, sections were rehydrated through absolute ethanol, 95% ethanol, 85% ethanol, 70% ethanol, and distilled water. They were then stained in 1% safranin for 4–6 h, gently rinsed with running water or distilled water, and differentiated through graded ethanol. Counterstaining was performed with 0.5% fast green for 30–60 s. After counterstaining, slides were rapidly dehydrated in 95% ethanol and absolute ethanol, cleared in xylene, and mounted with neutral balsam (Sinopharm Chemical Reagent Co., Ltd., Shanghai, China).

Microscopic observation of histological sections was performed using the IX73 microscope and DP75 digital camera system described above. For each sample type, at least three biological replicates were selected, and no fewer than five representative sections were examined per replicate. Typical histological features of normal prothalli, filamentous-arrest materials, thick fan-shaped blades, irregular spatulate blades, and the different archegonial developmental stages were recorded.

Histological observations in this study were used for qualitative classification and illustration. No morphometric variables were quantified from the section images; this limitation is explicitly considered in the Discussion.

### 4.4. Classification and Scoring Criteria for Developmental Disorders

Developmentally abnormal gametophytes were classified according to external morphology and histological structure. Filamentous arrest was defined as persistent wool-like or filamentous growth without stable two-dimensional lamellar expansion. Thick fan-shaped blades were defined as conspicuously enlarged, thickened, fan-shaped, or clump-like blades with compact internal tissue and poor formation of a regular cordate notch. Irregular spatulate blades were defined as narrow, asymmetric, spoon-, or tongue-shaped gametophytes with unstable tissue polarity, often accompanied by local swelling or bending. Normal cordate gametophytes with a regular outline, a central wing-like region, and mature or nearly mature archegonia were used as developmentally competent controls.

For the medium-response experiment, developmental quality was operationalized by four measured indicators rather than by a single composite score: arrest rate and hypertrophy rate represented adverse developmental outcomes, whereas sporophyte initiation rate and mean young-sporophyte height represented favorable outcomes. Better developmental performance was inferred only when the adverse indicators decreased and the favorable indicators increased; no unvalidated aggregate index was calculated.

### 4.5. Medium Treatments, Experimental Design, and Index Measurement

The preliminary recovery experiment for disordered gametophytes included three material groups—subculture + hormones, hormone-free subculture, and primary hormone-free culture—with 11 treatments in total (Table 4). The formulation of Murashige and Skoog (MS) medium followed the original description [21]. Full-strength MS medium was used as the basal medium and 1/2 MS as the reduced-salt medium. When activated carbon was added, its concentration was 0.25 g/L. IAA, 6-BA (A8170), GA3 (G8910), activated carbon (A7750), sucrose, and agar (A8190) were obtained from Beijing Solarbio Science & Technology Co., Ltd. (Beijing, China). Basal media contained the appropriate MS or 1/2 MS inorganic salts, 10 g/L sucrose, and 7 g/L agar. The pH was adjusted to 5.8 before autoclaving at 121 °C for 20 min. Spore-derived starting materials were prepared using the disinfection and solid-culture procedure described in Section 4.1. Recovery cultures were maintained at 25 ± 1 °C under a 16 h light/8 h dark photoperiod. Each treatment included three independent biological replicates, and each replicate contained five culture bottles. Approximately 20 to 30 morphologically similar gametophytes were assessed per culture bottle. The mean values from the five bottles within each independent culture batch were employed as a statistical unit (*n* = 3 per treatment). Developmental recovery was evaluated after 60 days of treatment, and sporophyte initiation was documented after 90 days. Upon visible independent growth of a sporophyte, young-sporophyte height was measured from the basal attachment site to the apex of the tallest juvenile leaf.

### 4.6. Statistical Analysis

Percentage data were first calculated at the culture-bottle level and then averaged within each independent biological replicate. Arrest rate was calculated as the number of gametophytes showing filamentous arrest divided by the total number evaluated × 100%. Hypertrophy rate was calculated as the number of thick fan-shaped or clump-like gametophytes divided by the total number evaluated × 100%. Sporophyte initiation rate was calculated as the number of gametophytes producing at least one visible young sporophyte with a young leaf divided by the total number evaluated × 100%. Young-sporophyte height was analyzed using replicate means. Normality and homogeneity of variance were evaluated using the Shapiro–Wilk test and Levene test, respectively. When parametric assumptions were met or approximately met, one-way ANOVA followed by Duncan multiple-range tests was used. The Kruskal–Wallis nonparametric test was applied as a robustness check. Pearson and Spearman correlation analyses were used to assess relationships among developmental indicators. The significance threshold was set at *p* < 0.05. Statistical analyses were performed using IBM SPSS Statistics 27.0 (IBM Corp., Armonk, NY, USA), and statistical graphs were prepared using OriginPro 2024 (OriginLab Corporation, Northampton, MA, USA).

### 4.7. Figure Preparation and Image-Quality Control

All microscopic and histological images were assembled as multi-panel figures with consistent panel-letter labels. Scale bars were calibrated and retained in each panel using ImageJ 1.54t (National Institutes of Health, Bethesda, MD, USA). Arrows or arrowheads were used to indicate key structures, including rhizoids, antheridia, archegonial neck cells, the venter region, egg cells, and abnormal cytological features.

## 5. Conclusions

Within the limits of morphological observation, qualitative histology, and comparative medium-response data, *Cibotium barometz* gametophyte abnormalities can be operationally classified as filamentous arrest, thick fan-shaped blades, and irregular spatulate blades. These phenotypes are consistent with, but do not prove, putative bottlenecks involving the one-dimensional-to-two-dimensional transition, a vegetative/storage bias, and unstable tissue polarity, respectively. Medium composition was significantly associated with quantified developmental outcomes. Hormone-free one-half-strength Murashige and Skoog (1/2 MS) medium supplemented with 0.25 g/L activated carbon produced the most favorable combination of low arrest and hypertrophy, high sporophyte initiation, and greater young-sporophyte height under the tested conditions. The study therefore provides a candidate diagnosis–recovery–initiation workflow and a quantitative outcome framework for *Cibotium barometz* in vitro propagation. It does not establish the underlying molecular, hormonal, or activated-carbon adsorption mechanisms; quantitative histology, gene-expression validation, endogenous-hormone profiling, and chemical analysis of the culture medium are required before causal conclusions are drawn.

## Figures and Tables

**Table 1:** Developmental timing and diagnostic criteria from spore germination to sporophyte formation in *Cibotium barometz*.

Stage	Time	Main Features	Diagnostic Meaning
Spore imbibition	3–15 d	Spores absorb water, swell and become lighter in color.	Initiation of germination
Spore-wall rupture	10–25 d	The spore-wall cracks and the cellular contents protrude.	Criterion for true germination
Rhizoid emergence	Approx. 20 d	A transparent or pale rhizoid emerges from the rupture site.	Basis for germination-rate scoring
Filamentous prothallus	30–45 d	Green cells divide in a one-dimensional filamentous pattern.	Risk window for developmental arrest
Lamellar prothallus	≥45 d	Division orientation changes and a two-dimensional blade is established.	Morphogenetic transition checkpoint
Cordate gametophyte	55–70 d	A notch, wing-like tissue and a gametangial region become visible.	Reproductive differentiation stage
Sporophyte formation	>70 d	Young leaves or roots appear and autonomous growth begins.	Evaluation of sporophyte initiation efficiency

**Table 2:** Developmental stages and paraffin-section criteria for archegonial ontogeny in *Cibotium barometz*.

Stage	Section Features	Developmental Interpretation
Initial-cell stage	Local vegetative cells enlarge; neck and venter are not yet differentiated.	Initiation of archegonial fate
Primordium stage	A compact cell cluster with dense cytoplasm is formed.	Establishment of the archegonial primordium
Early neck-cell differentiation	A preliminary neck outline appears and cells begin longitudinal alignment.	Beginning of structural regionalization
Late neck-cell differentiation	Neck cells elongate and become tightly arranged; venter cells enlarge.	Pre-maturation organization
Mature stage	A flask-shaped archegonium is visible, and the egg cell can be distinguished.	Potential for fertilization
Pre-fertilization stage	The neck canal is distinct and the venter remains stable.	Fertilization window

**Table 3:** Morphological and qualitative histological classification of normal and developmentally disordered gametophytes, with working interpretations requiring quantitative and molecular validation.

Type	External Morphology	Histological Features	Working Interpretation
Normal prothallus	Green, cordate, or nearly cordate, with a regular outline	Regular cell arrangement; clear cell boundaries and nuclei; mature archegonia can form	Developmentally competent control based on morphology and histology
Filamentous arrest	Wool-like or filamentous mass; no obvious two-dimensional blade	Persistent filamentous cells; single division orientation; absence of lamellar expansion	Pattern consistent with failure of the 1D-to-2D morphogenetic transition
Thick fan-shaped blade	Large, thick, fan-shaped, or clump-like blade	Dense elongated cells; starch-like storage accumulation; local wall thickening or lignification-like staining	Pattern consistent with vegetative/storage bias and reduced reproductive competence
Irregular spatulate blade	Narrow, asymmetric, or spoon-shaped blade with local swelling	Disordered polarity; blurred nuclear membrane or multinucleate-like cells; unstable archegonial initials	Pattern suggestive of unstable differentiation or polarity; molecular validation is required

**Table 4:** Effects of medium treatments on developmental recovery of *Cibotium barometz* gametophytes.

ID	Group	Treatment	Arrest (%)	Hypert. (%)	Init. (%)	Height (cm)
T1	Sub. + horm.	MS + IAA + 6-BA + AC	43.88 ± 5.30 ^a^	12.19 ± 2.90 ^d^	25.85 ± 2.63 ^e^	1.27 ± 0.27 ^g^
T2	Sub. + horm.	1/2MS + GA_3_ + 6-BA	10.80 ± 1.92 ^d^	16.85 ± 2.53 ^c^	62.56 ± 7.23 ^b^	2.82 ± 0.50 ^d^
T3	Sub. + horm.	MS	7.29 ± 2.58 ^d^	57.12 ± 4.34 ^a^	18.32 ± 2.11 ^e^	0.91 ± 0.20 ^h^
T4	Hormone-free subculture	MS	46.65 ± 3.91 ^a^	22.53 ± 2.72 ^b^	12.43 ± 2.55 ^g^	0.85 ± 0.18 ^h^
T5	Hormone-free subculture	MS + AC	34.74 ± 4.90 ^b^	14.29 ± 2.31 ^c^	9.35 ± 1.83 ^g^	0.97 ± 0.37 ^g^
T6	Hormone-free subculture	1/2MS	24.29 ± 3.08 ^c^	17.75 ± 2.96 ^c^	22.61 ± 2.55 ^e^	1.78 ± 0.43 ^f^
T7	Hormone-free subculture	1/2MS + AC	7.83 ± 2.30 ^d^	5.26 ± 1.13 ^e^	71.45 ± 9.16 ^a^	3.52 ± 0.61 ^b^
T8	Primary hormone-free	MS	37.56 ± 3.43 ^b^	25.01 ± 3.08 ^b^	18.99 ± 2.05 ^f^	1.58 ± 0.29 ^f^
T9	Primary hormone-free	MS + AC	22.31 ± 3.59 ^c^	12.33 ± 2.45 ^d^	35.24 ± 5.02 ^d^	2.54 ± 0.57 ^e^
T10	Primary hormone-free	1/2MS	17.71 ± 2.33 ^c^	9.91 ± 0.75 ^d^	47.19 ± 5.58 ^c^	2.97 ± 0.48 ^c^
T11	Primary hormone-free	1/2MS + AC	5.00 ± 0.31 ^e^	5.00 ± 0.31 ^e^	70.54 ± 9.07 ^a^	4.16 ± 0.72 ^a^

Note: Data are expressed as mean ± standard deviation. Within the same column, different lowercase letters denote statistically significant differences among treatments (*p* < 0.05). Independent culture batches (*n* = 3) served as the statistical units, with five replicate bottles per treatment within each batch. Abbreviations: Sub., subculture; horm., hormones; Hypert., hypertrophy; Init., sporophyte initiation; AC, activated carbon; Height, mean young-sporophyte height (cm). Arrest, hypertrophy, and sporophyte initiation values are percentages (%). Hormone concentrations were 1.0 mg/L IAA and 0.5 mg/L 6-BA in T1, and 0.04 mg/L GA_3_ and 0.02 mg/L 6-BA in T2; AC was 0.25 g/L.

## Data Availability

The data supporting the findings of this study, including replicate-level scoring data from the medium-regulation experiment, are available from the corresponding author upon reasonable request.
